# Correction of Excyclotropia by Surgery on the Inferior Rectus Muscle in Patients with Thyroid Eye Disease: A Retrospective, Observational Study

**DOI:** 10.1371/journal.pone.0159562

**Published:** 2016-07-19

**Authors:** Yasuhiro Takahashi, Yoshiyuki Kitaguchi, Shunsuke Nakakura, Hidenori Mito, Akiko Kimura, Hirohiko Kakizaki

**Affiliations:** 1 Department of Oculoplastic, Orbital & Lacrimal Surgery, Aichi Medical University Hospital, Aichi, Japan; 2 Department of Ophthalmology, Saneikai Tsukazaki Hospital, Himeji, Japan; 3 Ide Eye Hospital, Yamagata, Japan; 4 Department of Ophthalmology, Hyogo College of Medicine, Hyogo, Japan; University of Birmingham, UNITED KINGDOM

## Abstract

**Purpose:**

To examine the characteristics of excyclotropia correction through surgery on the inferior rectus muscle in patients with thyroid eye disease.

**Methods:**

This was a retrospective, observational study at a single institution. We reviewed 36 patients who had undergone unilateral inferior rectus muscle recession, with or without nasal inferior rectus muscle transposition. The following factors were investigated as possibly influencing excyclotropia correction: inferior rectus muscle thickness, degree of adipose change in the inferior rectus muscle, smoking status, history of orbital radiotherapy, and the amount of inferior rectus muscle recession. Using T1-weighted coronal magnetic resonance imaging, we measured the cross-sectional area of the inferior rectus muscle at its largest point, as well as the bright-signal area of the inferior rectus muscle, which reflects intermuscular adipose change. We then calculated the percentage internal bright-signal area at the point of the largest inferior rectus muscle cross-sectional area. The history of orbital radiotherapy was graded using a binary system. We evaluated correlations among excyclotropia correction, the amount of nasal inferior rectus muscle transposition, and the possible influencing factors listed, using stepwise multiple regression analyses.

**Results:**

The multiple regression model demonstrated a significant relationship among excyclotropia correction, amount of nasal inferior rectus muscle transposition, and the amount of inferior rectus muscle recession (Y_CORRECTION_ = 8.546X_TENDON WIDTH_ + 0.405X_RECESSION_− 0.908; r = 0.844; adjusted r^2^ = 0.695; *P* < 0.001).

**Conclusions:**

Excyclotropia correction was correlated with the amount of nasal inferior rectus muscle transposition and the amount of inferior rectus muscle recession, but not with the other factors. The regression model presented in this study will enable us to determine more precisely the amount of nasal inferior rectus muscle transposition in patients with excyclotropia of various angles.

## Introduction

Excyclotropia impairs binocular single vision and has a strong negative impact on patients’ daily activities [1]. This visual disorder is commonly accompanied by thyroid-associated inferior rectus (IR) myopathy with fibrotic contracture [2–5]. Patients can compensate for mild excyclotropia by head tilt [3–6], but severe forms of the disorder cannot be completely compensated in this manner; in addition, excyclotropia cannot be corrected using prisms [7].

Nasal transposition of the fibrous IR muscle is a reasonable treatment for excyclotropia in thyroid eye disease (TED) [8–10]. One previous study reported a 7° excyclotropia that was corrected using nasal transposition of one IR muscle tendon width [8]. However, the researchers who performed the study did not analyze the correlation between the angle of excyclotropia and the amount of nasal IR muscle transposition [8]. It is therefore unclear whether a dose–effect relationship can be extrapolated to patients with different degrees of excyclotropia.

The outcomes of strabismus surgery in patients with TED are less reproducible than those in patients without TED [11]. Therefore, to ensure a more tailored surgery, it is vital that clinicians understand the factors that influence strabismus in patients with TED. Presumptive influential factors for strabismus surgery in patients with TED include thickness of the target extraocular muscles, severity of adipose change in the transposed muscles, smoking status, histories of orbital radiotherapy and orbital decompression surgery, and the amount of concomitant muscle recession [11–20]. The thickness of the extraocular muscles and adipose changes reflect the severity of fibrous changes therein [18]. Smoking and orbital radiotherapy lead to excessive orbital fibrosis [3,19]. Orbital decompression surgery restricts extraocular muscle motility [20]. Muscle recession releases a fibrous contracture of the extraocular muscle [13]. However, no previous studies have shown the influences of these factors on excyclotropia correction in patients with TED.

In this study, we analyzed the correlations among excyclotropia correction, the amount of nasal IR muscle transposition, and the aforementioned influencing factors in patients with TED.

## Materials and Methods

### Patients

This study was a retrospective chart review of all patients with TED who underwent unilateral IR muscle recession with nasal IR muscle transposition, performed by one of the authors (H.K.), between March 2011 and December 2015. We also reviewed the medical records of all patients with TED who underwent unilateral simple IR muscle recession, carried out by the same surgeon, during the same period. Five patients underwent bilateral IR muscle recession during the study period, and a further seven patients underwent combination surgery of IR muscle recession on the affected side and superior rectus muscle recession on the unaffected side. However, we excluded these data from the analyses, because they were influenced by the concomitant procedure, and we wished to assess the effect of isolated unilateral surgery.

This study was approved by the Institutional Review Board (IRB) of Aichi Medical University Hospital (no. 15–025), and followed the tenets of the Declaration of Helsinki. The IRB granted a waiver of informed consent for this study on the basis of the ethical guidelines for medical and health research involving human subjects established by the Japanese Ministry of Education, Culture, Sports, Science and Technology, and by the Ministry of Health, Labour, and Welfare. The waiver was granted because the study was a retrospective chart review, not an interventional study, and because it was difficult to obtain consent from patients who had been treated several years prior. Nevertheless, at the request of the IRB, we published an outline of the study, available for public viewing, on the Aichi Medical University website; this also gave patients the opportunity to decline participation. None of the patients declined participation. Personal identifiers were removed from the records prior to data analyses.

A diagnosis of TED was the presence of at least one of the characteristic eyelid signs (eyelid fullness, eyelid retraction, and/or eyelid lag) as well as the presence of thyroid autoimmunity [21]. The inferior rectus muscle enlarged without muscle tendon involvement in all patients, which also supported the diagnosis of TED [22]. All patients included in the study were controlled as euthyroid at the time of surgery, and were in the static or chronic “burnout” phase of TED. We judged the condition of each patient’s disease using the clinical activity score (CAS) [23], as well as by the presence or absence of inflammation in the extraocular muscles, as revealed by magnetic resonance imaging (MRI). The CAS was calculated using seven parameters: retrobulbar discomfort, pain on eye movement, eyelid erythema, eyelid swelling, conjunctival injection, chemosis, and swollen caruncle [23]. When the patient had a CAS of less than three points, their TED was defined as being in the static or chronic “burnout” phase [23].

The exclusion criteria were a history of strabismus surgery, temporal IR muscle transposition, missing clinical data, and a follow-up time of < 3 months. We also excluded patients who had an intracranial lesion revealed on MRI and those with concomitant neuro-ophthalmologic disorder(s). We could not statistically evaluate the influence of the history of orbital decompression surgery on excyclotropia correction, because only one patient had applicable data. For this reason, we excluded this patient from this study.

### Data Collection

The following data were collected: age, sex, CAS, surgical side, MRI findings, smoking status, history of orbital radiotherapy, amounts of nasal IR muscle transposition and IR muscle recession, preoperative and postoperative angles of ocular deviation, and preoperative and postoperative binocular single vision fields.

### Magnetic Resonance Imaging

MRI was performed using a 1.5-Tesla scanner (Magnetom Abant™; Siemens Healthcare, Erlangen, Germany), with the patients in the supine position. Coronal T1- and T2-weighted gradient-echo sequences were acquired (T1—repetition time: 500 ms, echo time: 10 ms, field of view: 140 × 140 mm, matrix: 256 × 220, section thickness: 3 mm with a 0.6 mm gap between slices; T2—repetition time: 4000 ms, echo time: 100 ms, all other parameters were the same as in T1). Patients were asked to look at a light source to ensure their eyes were fixed in the primary position.

The IR muscle cross-sectional area at the largest point was measured on a T1-weighted MRI image by one of the authors (Y.T.), using the measuring tool available in the MRI viewer (ShadeQuest/ViewR™; Yokogawa Medical Solutions Corporation, Tokyo, Japan). The same author also examined the internal bright-signal in the IR muscle, which reflects adipose change within the muscle. The high intensity area was measured on a T1-weighted MRI image at the largest IR cross-sectional area, using the aforementioned measuring tool (Fig 1). We calculated the percentage internal bright-signal area at the largest IR muscle thickness as follows: the internal bright-signal area/the largest IR muscle thickness × 100.

**Fig 1:**
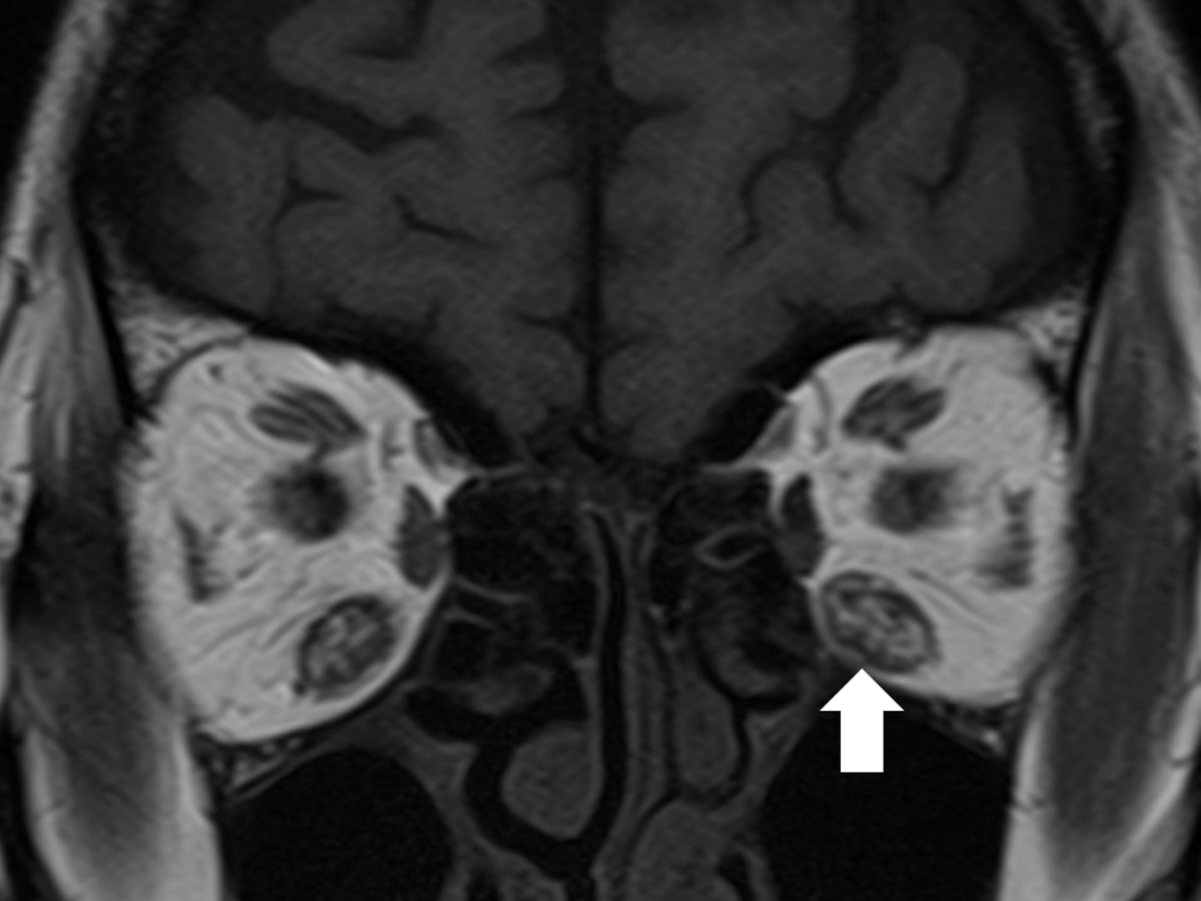
Internal bright signal in the inferior rectus muscle. A high intensity area in the inferior rectus muscle is measured on a T1-weighted coronal magnetic resonance image (arrow).

### Smoking Status

We asked all patients the number of cigarettes they smoked per day. All smokers were current smokers at the first examination, while all non-smokers had never smoked.

### History of Orbital Radiotherapy

The dose used for orbital radiotherapy was 20 Gy in all treated patients. Previous orbital radiotherapy was expressed using a binary system (a dummy variable; 0 = with no history, 1 = with a history) [24].

### Orthoptic Examination

The angle of ocular deviation was measured using a synoptophore (Clement Clarke International Ltd., Edinburgh, UK) 1 day before surgery and 3 months after surgery. The patient's head was positioned upright, and the instrument set such that the fixating eye was the eye on which surgery was planned. One of two arms of the synoptophore was fixed at 0°. We used two slides: a black circle with a cross-shaped blank and a black cross (L-25G; Inami, Tokyo, Japan) (Fig 2). The black circle slide was fixed in the arm of the fixating eye, and the patient was asked to move the black cross until it was positioned appropriately within the circle. We recorded the angle at which this was achieved.

**Fig 2:**
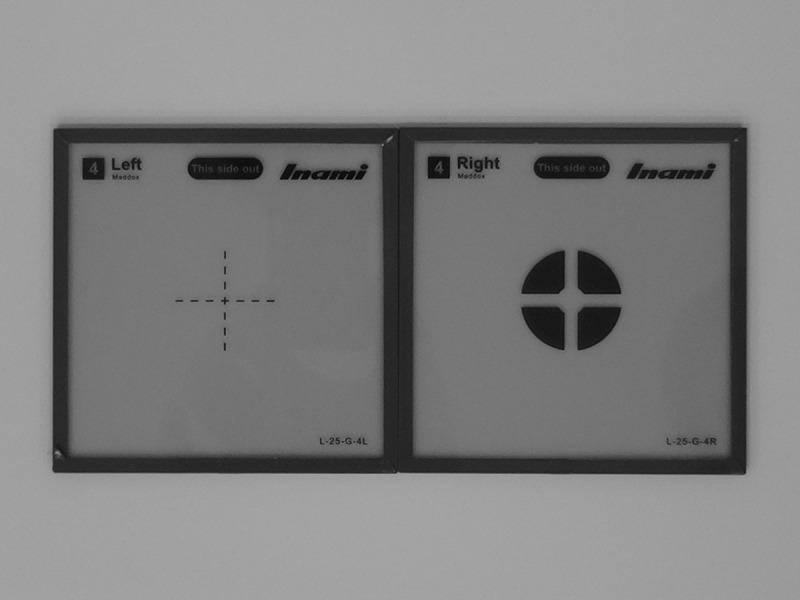
Slides for the synoptophore. A black cross (left) and a black circle with a cross-shaped blank (right) were used.

### Surgical Technique

Surgery was performed under either general or local anesthesia. A perilimbal conjunctival incision, with radial relaxing incisions, was made in the inferior quadrant. A muscle hook was used to secure the IR muscle at its insertion, and the Tenon’s capsule around the IR muscle was thoroughly dissected using cotton swabs. As the tendon width varies between individuals [25], the width of the IR muscle tendon was measured at the scleral insertion using a caliper. The IR muscle tendon was secured using locking 8–0 polyglactin sutures at two points (Vicryl^®^; Johnson and Johnson Company, New Brunswick, NJ, USA) 1 mm posterior to the globe insertion, because the tip thickness of the muscle hook was 1 mm. Next, the IR muscle was detached from its insertion. In patients with simple IR muscle recession, the sutures were fixed to the sclera 1 mm posterior to the point that was estimated on the basis of the preoperative ocular deviation angle. Recession of the IR muscle was calculated as follows: 2° of ocular deviation per 1 mm IR muscle recession [8,26]. In patients with both IR muscle recession and nasal IR muscle transposition, we transposed the IR muscle nasally along the spiral of Tillaux; we did so concomitantly with IR muscle recession. The amount of nasal IR muscle transposition was preoperatively calculated based on the preoperative deviation angle and the measurement result of the tendon width as follows: 7° of ocular deviation per one IR muscle tendon width transposition, assuming that the previously reported dose–effect relationship correlated linearly [8]. The IR muscle tendon was additionally fixed to the sclera using 8–0 polyglactin sutures at two to four points to prevent slippage of the IR muscle. Finally, the conjunctiva was closed using 8–0 polyglactin sutures.

### Statistical Analyses

Patient age, IR muscle cross-sectional area, percentage internal bright-signal area at the point of largest IR muscle thickness, and number of cigarettes smoked per day were expressed as means ± standard deviations. In patients with simple IR muscle recession, the angle of cyclotropia before surgery was compared with that after surgery using the paired *t*-test. The correlations among excyclotropia correction, nasal IR muscle transposition, and the aforementioned hypothesized factors were analyzed using stepwise multiple regression analyses. All statistical analyses were performed using SPSS™ version 22 software (IBM Japan, Tokyo, Japan). A *P-*value of < 0.05 was considered statistically significant.

## Results

Thirty-six patients (13 men, 23 women; mean age, 60.3 ± 10.4 years; range, 38–78 years; 17 right eyes, 19 left eyes) were included in this study.

The largest IR muscle cross-sectional area was 79.8 ± 21.4 mm^2^ (range, 41.9–136.4 mm^2^). The percentage internal bright-signal area at the point of largest IR muscle thickness was 22.6% ± 22.2% (range, 0%–81.4%). Twenty-five patients (6 men, 19 women) were non-smokers. The mean number of cigarettes smoked per day in smokers was 3.8 ± 6.7 (range, 3–20 cigarettes per day). Orbital irradiation was performed in 14 patients (6 men, 8 women).

The IR muscle was nasally transposed in 25 patients (69.4%), while the remaining 11 patients (30.6%) underwent simple IR muscle recession. Based on clinical evidence, none of the patients developed a slipped IR muscle postoperatively. All patients obtained binocular single vision at the primary position after surgery. Of the 11 patients with simple IR muscle recession, excyclotropia was reduced in 8 patients (72.7%; mean, 2.4°; range, 1°–5°). This reduction was statistically significant (*P* = 0.003). The mean reduction in excyclotropia was 3.0° in three patients with a 1/4 IR muscle tendon width transposition (range, 3°–3°), 4.3° in nine patients with a 1/2 IR muscle tendon width transposition (range, 3°–8°), 8.0° in two patients with a 2/3 IR muscle tendon width transposition (range, 7°–9°), 5.2° in five patients with a 3/4 IR muscle tendon width transposition (range, 2°–8°), and 11.7° in six patients with a one IR muscle tendon width transposition (range, 8°–15°).

A multiple regression formula showed that excyclotropia, amount of nasal IR muscle transposition, and amount of concomitant IR muscle recession were significantly correlated (Y_CORRECTION_ = 8.546X_TENDON WIDTH_ + 0.405X_RECESSION_− 0.908; r = 0.844; adjusted r^2^ = 0.695; *P* < 0.001). The coefficient of the ‘X’ variable indicated a positive correlation with excyclotropia correction. By a stepwise process, IR muscle thickness, the internal bright-signal area, smoking status, and a history of orbital radiotherapy were removed from the regression model.

## Discussion

In the current study, multiple regression analyses identified a significant relationship among excyclotropia correction, the amount of nasal IR muscle transposition, and the amount of concomitant IR muscle recession in patients with TED. IR muscle thickness, the internal bright-signal area, smoking status, and a history of orbital radiotherapy showed no such correlation.

Correction of the excyclotropia was positively correlated with the amount of the nasal IR muscle transposition. The formula presented in this study will enable clinicians to determine more precisely the amount of nasal IR muscle transposition in patients with excyclotropia of various angles.

Excyclotropia was significantly reduced after a simple IR muscle recession in 8 of 11 patients (72.7%; mean, 2.4°; range, 1°–5°). A previous study demonstrated a similar result; namely, that excyclotropia decreased after simple IR muscle recession in all five patients with TED (range, 4–8 prism diopters) [27]. Taken together, these results indicate that simple IR muscle recession can correct mild excyclotropia in patients with TED. Because fibrous contracture of the IR muscle causes excess excycloduction, IR muscle recession decreases excycloduction [13].

The thickness of the IR muscle, the internal bright-signal within the IR muscle, and smoking status did not correlate with excyclotropia correction, even though these factors are associated with the severity of fibrotic contracture in the IR muscle [3,18,19]. This indicates that these factors are not predictors of excyclotropia correction.

A history of orbital radiotherapy did not significantly affect the correlation between nasal IR muscle transposition and excyclotropia correction. This was similar to our previous study, which demonstrated no correlation between the effect of IR muscle recession and a history of orbital radiotherapy [18]. Even though orbital radiotherapy leads to orbital fibrosis, it decreases thyroid-related orbital inflammation, which may offset the radiation-induced orbital fibrosis [18].

The coefficient of the tendon width in the regression model was 8.546, and the mean excyclotropia correction in the six patients with one IR muscle tendon width transposition was 11.7°. The excyclotropia correction in this study appeared to be larger than that in the previous study mentioned (7° of excyclotropia correction by nasal transposition per one IR muscle tendon width), although all patients in both studies obtained binocular single vision at the primary position [8]. Differences between the present (surgical eye) and previous studies (non-surgical eye) in terms of the position of the fixing eye during the synoptophore test may account for this discrepancy [8,28]. When the surgical eye is fixed during the synoptophore test, a larger deviation angle may be estimated by Hering’s law than when the non-surgical eye is fixed [29].

Inferior oblique muscle weakening surgery can be used for excyclotropia correction [30]. However, nasal transposition of an affected IR muscle is a more reasonable, easier, and more quantitative procedure [30]. In addition, excyclotropia is commonly accompanied by thyroid-associated IR myopathy with fibrotic contracture [2–5], which also causes hypotropia. The combination of nasal transposition and recession of the IR muscle can simultaneously correct excyclotropia and hypotropia.

The main methodological limitation of the present study was its retrospective design. In addition, we included patients with TED who did not have a history of orbital decompression surgery. Orbital decompression surgery causes more restriction of extraocular muscle motility [20]. Therefore, the present regression model cannot be applied to patients with TED who have a history of orbital decompression surgery. Future studies are necessary to determine regression models for such patients.

In conclusion, excyclotropia correction in patients with TED was correlated with the amount of the nasal IR muscle transposition and the amount of concomitant IR muscle recession, but not with IR muscle thickness, internal bright-signal area, smoking status, and a history of orbital radiotherapy.

## Supporting Information

S1 File Data analyzed. (XLSX)

## References

[1] Kimura A. Management of thyroid-associated ophthalmopathy: strabismus surgery: Part 2. Rinsho Ganka 2013;67:1630–1636 (Japanese).

[2] Nardi M. Eye muscle surgery In: Wiersinga WM, Kahaly GJ, editors. Graves’ Orbitopathy: A Multidisciplinary Approach. Basel: Karger; 2007 pp. 176–187.

[3] Nardi M. Squint surgery in TED: Hints and fints, or why Graves’ patients are difficult patients. Orbit 2009;28:245–250. 19839883 10.1080/01676830903104603

[4] Trobe JD. Cyclodeviation in acquired vertical strabismus. Arch Ophthalmol 1984;102:717–720. 6721761 10.1001/archopht.1984.01040030573021

[5] Caygill WM. Excyclotropia in dysthyroid ophthalmopathy. Am J Ophthalmol 1972;73:437–441. 5013251 10.1016/0002-9394(72)90073-6

[6] Chen VM, Dagi LR. Ocular misalignment in Graves’ disease may mimic that of superior oblique palsy. J Neuro-Ophthalmol 2008;28:302–304. 10.1097/WNO.0b013e31818f133e 19145130

[7] Woo SJ, Seo JM, Hwang JM. Clinical characteristics of cyclodeviation. Eye 2005;19:873–878. 15731772 10.1038/sj.eye.6701675

[8] Kimura A. Management of thyroid associated ophthalmopathy: strabismus surgery: Part 1. Rinsho Ganka 2013;67:1452–1457 (Japanese).

[9] Kushner BJ. Torsion and pattern strabismus: Potential conflicts in treatment. JAMA Ophthalmol 2013;131:190–193. 10.1001/2013.jamaophthalmol.199 23411884

[10] von Noorden GK, Chu MW. Surgical treatment options in cyclotropia. J Pediatr Ophthalmol Strabismus 1990;27:291–293. 2086744 10.3928/0191-3913-19901101-05

[11] Nassar MM, Dickinson AJ, Neoh C, Powell C, Buck D, Galal E, et al Parameters predicting outcomes of strabismus surgery in the management of Graves’ ophthalmopathy. J AAPOS 2009;13:236–240. 10.1016/j.jaapos.2008.11.007 19395292

[12] Peragallo JH, Velez FG, Demer JL, Pineles SL. Postoperative drift in patients with thyroid ophthalmopathy undergoing unilateral inferior rectus muscle recession. Strabismus 2013;21:23–28. 10.3109/09273972.2012.762533 23477773 PMC3714169

[13] Jellema HM, Saeed P, Everhard-Halm Y, Prick L, Mourits M. Bilateral inferior rectus muscle recession in patients with Graves’ orbitopathy: Is it effective? Ophthal Plast Reconstr Surg 2012;28:268–272. 10.1097/IOP.0b013e3182565c26 22689133

[14] De Hoog J, Stravers S, Kalmann R. Recession of the inferior rectus muscle in Graves’ orbitopathy. Eye 2010;24:1011–1017. 10.1038/eye.2009.267 19911020

[15] Pitz S, Esch A, Müller-Forell WS, Kahaly G, Mann W, Pfeiffer N. Is there a relationship between the degree of preoperative motility impairment of the muscle thickness and the outcome of strabismus surgery in patients with Graves’ orbitopathy after decompression surgery? Orbit 2005;24:173–176. 16169802 10.1080/01676830500182705

[16] Yoo SH, Pineles SL, Goldberg RA, Velez FG. Rectus muscle resection in Graves’ ophthalmopathy. J AAPOS 2013;17:9–15. 10.1016/j.jaapos.2012.09.018 23352720 PMC3715128

[17] Mourits MP, Koorneef L, van Mourik-Noordenbos AM, van der Meulen-Schot HM, Prummel MF, Wiersinga WM, et al Extraocular muscle surgery for Graves’ ophthalmopathy: does prior treatment influence surgical outcome? Br J Ophthalmol 1990;74:481–483. 2390524 10.1136/bjo.74.8.481 PMC1042178

[18] Takahashi Y, Kakizaki H. Predictors of the dose-effect relationship regarding unilateral inferior rectus muscle recession in patients with thyroid eye disease. Int J Endocrinol 2015; 2015:703671 10.1155/2015/703671 26221140 PMC4499406

[19] Prendiville P, Chopra M, Gauderman WJ, Feldon SE. The role of restricted motility in determining outcomes for vertical strabismus surgery in Graves’ ophthalmopathy. Ophthalmology 2000;107:545–549. 10711893 10.1016/s0161-6420(99)00145-1

[20] Gilbert J, Dailey RA, Christensen LE. Characteristics and outcomes of strabismus surgery after orbital decompression surgery. J AAPOS 2005;9:26–30. 15729277 10.1016/j.jaapos.2004.10.004

[21] Mourits MP. Diagnosis and differential diagnosis of Grave’s orbitopathy In: Wiersinga MM, Kahaly GJ, editors. Grave’s Orbitopathy: A Multidisciplinary Approach. Basel: Karger; 2007 pp. 66–77.

[22] Kahaly GJ. Imaging in thyroid-associated orbitopathy. Eur J Endocrinol 2001;145:107–118. 11454505 10.1530/eje.0.1450107

[23] Mourits MP, Koornneef L, Wiersinga WM, Prummel MF, Berghout A, van der Gaag R. Clinical criteria for the assessment of disease activity in Grave’s ophthalmopathy: a novel approach. Br J Ophthalmol 1989;73:639–644. 2765444 10.1136/bjo.73.8.639 PMC1041835

[24] Skrivanek S. The use of dummy variables in regression analysis. Available: https://www.moresteam.com/whitepapers/download/dummy-variables.pdf.

[25] Dutton JJ. Extraocular muscles In: Dutton JJ, editor. Atlas of Clinical and Surgical Orbital Anatomy. 2nd edition. Philadelphia: Elsevier Saunders; 2011 pp. 29–49.

[26] Schittkowski M, Fichter N, Guthoff R. Strabismus surgery in Graves’ disease: dose-effect relationships and functional results. Klin Monbl Augenheilkd 2004;221:941–947. 15562358 10.1055/s-2004-813752

[27] Köse S, Üretmen Ö, Emre S, Pamukcu K. Recession of the inferior rectus muscle under topical anesthesia in thyroid ophthalmopathy. J Pediatr Ophthalmol Strabismus 2002;39:331–335. 12458843 10.3928/0191-3913-20021101-06

[28] Kubota M, Kimura A, Meguro C, Mahara C, Sano N, Tanaka M, et al Examination of the measurements of vertical and cyclo deviations in the patients who have vertical or cyclo strabismus. Japanese Orthoptic Journal 2008;37:91–94 (Japanese).

[29] von Noorden GK, Campos EC. Physiology of the ocular movements In: von Noorden GK, Campos EC, editors. Binocular Vision and Ocular Motility: Theory and Management of Strabismus. 6th edition. St. Louis: Mosby; 2002 pp. 52–84.

[30] Okamoto M, Kimura A, Masuda A, Mimura O. Surgical effects of nasal transposition of inferior rectus muscle: 135 cases of acquired superior oblique palsy. Clin Ophthalmol 2015:9 691–695. 10.2147/OPTH.S79482 25945030 PMC4407764

